# Seasonality and symptoms of depression: A systematic review of the literature

**DOI:** 10.1017/S2045796019000209

**Published:** 2019-04-22

**Authors:** Simon Øverland, Wojtek Woicik, Lindsey Sikora, Kristoffer Whittaker, Hans Heli, Fritjof Stein Skjelkvåle, Børge Sivertsen, Ian Colman

**Affiliations:** 1 Division of Mental and Physical Health, Norwegian Institute of Public Health, Bergen, Norway; 2 Department of Psychosocial Science, Faculty of Psychology, University of Bergen, Bergen, Norway; 3 Department of Psychological Medicine, Royal Infirmary of Edinburgh, Edinburgh, UK; 4 Health Sciences Library, University of Ottawa, Ottawa, Ontario, Canada; 5 The Research Institute, Modum Bad Psychiatric Center, Vikersund, Norway; 6 Lovisenberg Diaconal Hospital, Oslo, Norway; 7 Innlandet hospital trust, Norway; 8 Department of Mental Health, Norwegian University of Science and Technology, Trondheim, Norway; 9 Department of Research and Innovation, Helse Fonna HF, Haugesund, Norway; 10 School of Epidemiology & Public Health, University of Ottawa, Ottawa, Canada

**Keywords:** Admissions, antidepressants, depression, depressive symptoms, mood disorders, postpartum depression, seasonality, systematic review

## Abstract

**Aims:**

Lay opinions and published papers alike suggest mood varies with the seasons, commonly framed as higher rates of depression mood in winter. Memory and confirmation bias may have influenced previous studies. We therefore systematically searched for and reviewed studies on the topic, but excluded study designs where explicit referrals to seasonality were included in questions, interviews or data collection.

**Methods:**

Systematic literature search in Cochrane database, DARE, Medline, Embase, PsychINFO and CINAHL, reporting according to the PRISMA framework, and study quality assessment using the Newcastle-Ottawa scale. Two authors independently assessed each study for inclusion and quality assessment. Due to large heterogeneity, we used a descriptive review of the studies.

**Results:**

Among the 41 included studies, there was great heterogeneity in regards to included symptoms and disorder definitions, operationalisation and measurement. We also observed important heterogeneity in how definitions of ‘seasons’ as well as study design, reporting and quality. This heterogeneity precluded meta-analysis and publication bias analysis. Thirteen of the studies suggested more depression in winter. The remaining studies suggested no seasonal pattern, seasonality outside winter, or inconclusive results.

**Conclusions:**

The results of this review suggest that the research field of seasonal variations in mood disorders is fragmented, and important questions remain unanswered. There is some support for seasonal variation in clinical depression, but our results contest a general population shift towards lower mood and more sub-threshold symptoms at regular intervals throughout the year. We suggest future research on this issue should be aware of potential bias by design and take into account other biological and behavioural seasonal changes that may nullify or exacerbate any impact on mood.

## Introduction

Depression is common (Waraich *et al*., 2004) with reported 1-year prevalence estimates ranging around 6.6% in the USA (Kessler *et al*., 2003), 5.5% in Canada (Patten *et al*., 2015), 7.4% in Finland (Markkula *et al*., 2015) and is associated with significant disease burden worldwide (Whiteford *et al*., 2015). The causes and mechanisms behind depression are not fully understood but is commonly framed as a complex outcome of genetic, cognitive, behavioural and environmental risk factors operating in concert.

One of the environmental factors that continuously attracts attention from researchers and the public is how seasonal changes affects mood and depressive symptoms. Seasonal variations impact the prevalence and expression of certain diseases, with influenza serving as one example (Weinberger *et al*., 2012). A host of single studies suggest potential risk factors for depression may vary with seasons (Rosenthal *et al*., 1984; Roecklein and Rohan, 2005). For example, sleep patterns (Rosenthal *et al*., 1984; Lewy *et al*., 1987), levels of physical activity (Shephard and Aoyagi, 2009), reproductive behaviours (Roenneberg and Aschoff, 1990; Bronson, 1995), a host of neurobiological factors (Carlsson *et al*., 1980; Kivela *et al*., 1988; Avery *et al*., 1997; Neumeister *et al*., 2000; Lambert *et al*., 2002; Morera and Abreu, 2006; Kalbitzer *et al*., 2010; Abell *et al*., 2016) are reported to co-vary with seasonal variation and might impact on mood. However, the extent of this impact, and whether or not it translates to functional and clinical significance, remains controversial.

At the individual clinical level, some individuals report seasonal changes in mood that surpass thresholds of clinical significance (Rosenthal *et al*., 1984; Roecklein and Rohan, 2005). The label ‘seasonal affective disorder’ (SAD) emerged in the early 1980s to capture this phenomenon. Still, neither the ICD nor the DSM diagnostic system includes SAD as a distinct diagnosis. The DSM, since DSM-III-R, has included the possibility to specify if major depression or bipolar disorders occur in a seasonal pattern (Roecklein and Rohan, 2005). In ICD-11, seasonal pattern is now a specifier under mood disorders. The scientific controversy around the concept of SAD remains (Hansen *et al*., 2008; Traffanstedt *et al*., 2016; Young, 2017).

Mood is influenced by perceptions and psychosocial factors (Crum and Phillips, 2015). One study found that more people searched for depression-related terms on Internet-based search engines in winter (Ayers *et al*., 2013). This could be due to more people suffering from depression in winter, but possibly also a stronger focus on depression in media and peers during this time of year. Those processes may also reinforce each other, and an increased societal and media focus could make people attribute ambiguous symptoms to the season and depression during winter. Attribution sets are also likely to influence research on subjects' experience of seasonality and has relevance for the most commonly used measurement of seasonality, the Seasonal Pattern Assessment Questionnaire (SPAQ). The items in that questionnaire make the intent of measure seasonal variations in mood and behaviour explicit for the respondents. It, therefore, invites a mix of seasonal variation but also reports that reflect subjects' attributions of their symptoms. The questionnaire is criticised for this feature as it might invite memory and confirmation bias (Nayyar and Cochrane, 1996), and potentially lead to overestimation of seasonal effects. Furthermore, the reliability and validity of the SPAQ have been criticised (Mersch *et al*., 2004), and it is not considered a valid measurement of depression (Traffanstedt *et al*., 2016).

Knowing if, or how, depressive symptoms and mood fluctuate across seasons would contribute to an improved understanding of risk factors, mechanisms and epidemiology of depression. We therefore systematically reviewed the literature to examine if existing evidence supports the assumption of seasonal variation in the prevalence and symptoms of depression. Informed by the potential confirmation bias by self-report, we restricted our search to designs that circumvent this problem and asked, interviewed or collected data from participants without any explicit referral to seasonality as a topic of interest.

## Methods

### Literature search

We used a broad search strategy and selected the subset of papers on depression and depressive symptoms during the full-text paper review. The following databases were accessed as part of our search strategy: Cochrane Database of Systematic Reviews (via OVID), DARE (Database of Abstracts of Reviews of Effects via OVID), Cochrane Central Register of Controlled Trials (CENTRAL via OVID), Medline and Medline in Process (via OVID), Embase (via OVID), PsycINFO (via OVID) and the Cumulative Index to Nursing and Allied Health Literature (CINAHL via EBSCOHost). A search strategy was developed in consultation with a health sciences librarian (author LS) to identify keywords and Medical Subject Headings (MeSH) in Medline, which were then adapted for all other databases (see the Appendix). The search was conducted from the inception of each database to April 2015, with an updated search July 2017. There were no language exclusion criteria and no publication restrictions. All references were entered into Endnote for processing (*n* = 4393). After removal of duplicates, 2121 papers remained.

### Inclusion and exclusion

Papers were included based on the following:

*Type of study*. General population studies, registrybased studies, experimental studies and self-report studies published in peer-reviewed journals were considered for inclusion. We did not restrict papers on language or date of publication.

*Participants*. Youth and adults in the general population (i.e. animal studies and studies with children were excluded).

*Exposure*. Participants or the sample must have been exposed to more than one season individually or as a group.

*Comparison*. Repeated measurements over a year or enough measurements per month or per season to provide meaningful comparisons. Time-points had to be defined and presented in the paper. In studies where each participant was measured only once, other design features must have been in place to reasonably assume unbiased selection of time of measurement between subjects.

*Outcomes*. For the broader search, outcomes were defined as depressive symptoms, anxiety symptoms, symptoms of mental illness, depression, anxiety, mental illness, insomnia, sleep problems, sleep duration and -length, difficulties initiating sleep, suicidal thoughts, suicidal acts, self-harm, suicide, psychiatric hospital admissions. For the purpose of this paper, we focused on depression and depressive symptoms, and hospital admissions and prescriptions related to depression. Most studies on depression prevalence used a screening tool with case identification by the cut-off score. We accepted the authors' approach in these cases and labelled this ‘depression’ despite not having used a diagnostic interview schedule.

### Exclusion criteria

We excluded studies where the research hypothesis was available to the participants, or if the research hypothesis or variable measurement overtly related to seasonal variation. Due to these criteria, studies using the SPAQ or similar instruments eliciting the subjective experience of seasonality (Young *et al*., 2015) were excluded.

### Procedure

**Fig. 1.**
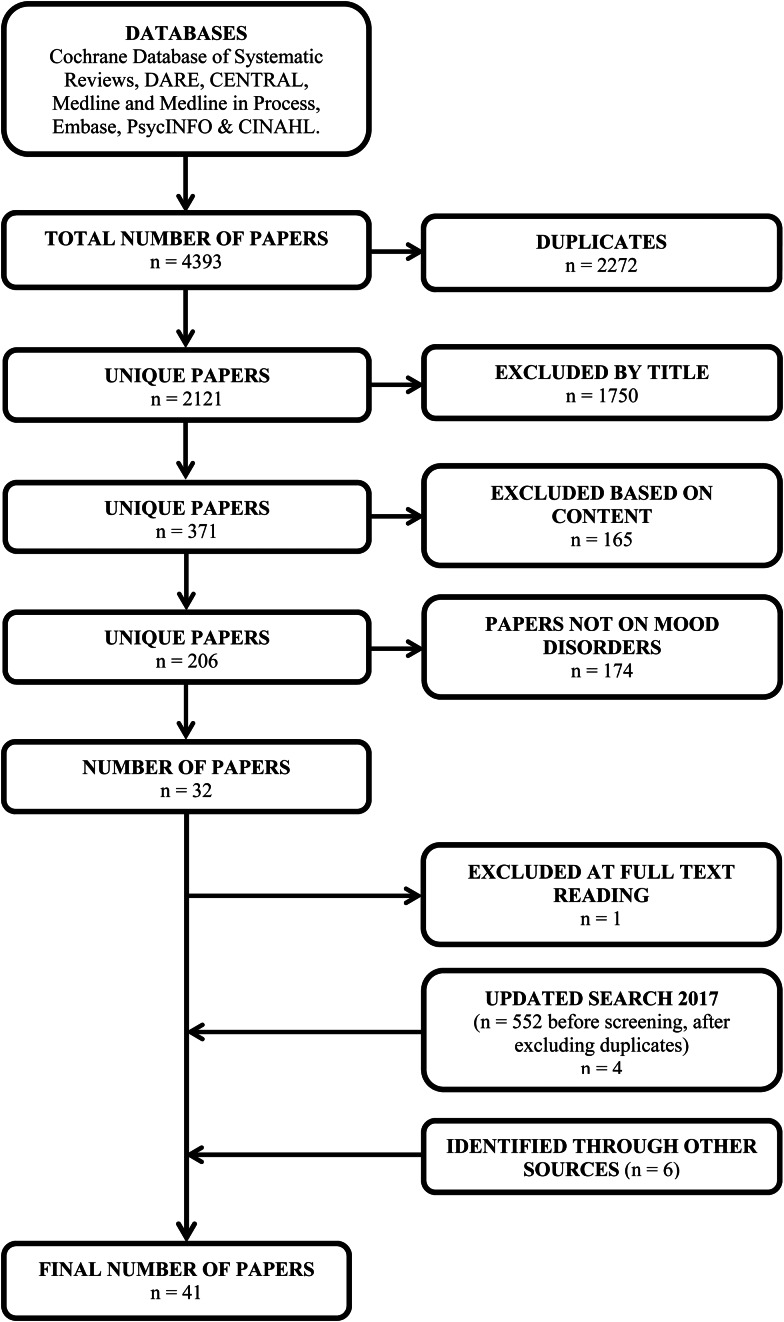
Flow diagram of the literature search and study exclusion process.

Title and abstract (if available) from the search was listed. The selection procedure (Fig. 1) from the initial papers were done in two rounds. First, two independent evaluators went through the list and excluded papers based on title and abstract, according to the inclusion and exclusion criteria. Disagreement in this phase led to the paper being included in the next round for full-text evaluation. In the next phase, the remaining papers were collected in full text and split into three separate lists. Two persons appraised each of the papers on the list against inclusion criteria. In case of disagreement, the third of this team of three was consulted to reach consensus. The reasons for disagreement were recorded. From the final set of papers, we selected those that had data on seasonal variation in depression. In July 2017, we updated the search following the same process as outlined for the main search and identified additional studies from other sources (ancestry approach).

### Study quality

Individual study quality and risk of bias were examined through the use of an adapted version of the Newcastle-Ottawa scale (NOS) (Wells *et al*., 2000). NOS is a tool to evaluate non-randomised studies. In its original form, it includes eight items across three dimensions: selection, comparability, and outcomes. Study quality is semi-quantified, with a maximum score of nine ‘stars’. The independent variable of interest in this study (seasons) leaves everyone exposed. There are therefore no non-exposed control groups in the studies. For this reason, we disregarded the second item of the scale (*selection of the non-exposed cohort*). Furthermore, as we were interested in the variability of depression over time (seasons), we excluded the fourth item of the scale (*demonstration that outcome of interest was not present at the start of study*) and were left with seven stars as the maximum.

### Summary measures

We expected and observed large degrees of heterogeneity in definitions, method of assessment, and summary measures amongst the included studies. Consequently, a meta-analysis of studies was not possible and a descriptive review follows.

## Results

Of the initial 2108 papers, 378 remained after title and abstract screening and were examined in full text. For the purpose of this review, a total of 32 papers were first included after exclusion by topic and study design (Fig. 1), one was discarded upon further examination of the full text. Another four papers were added after an updated literature search, and a total of six studies were identified through other papers and included. The final list comprised 41 papers (Table 1). Six and 18 studies got a high-quality rating with full score or only point deducted, respectively, using the adapted Newcastle-Ottawa rating scale (Table 2).

**Table 1.** Description and main findings of included studies

Author (Year)	Time period	Number of participants	Study origin	Design	Measurement	Measurement and outcome	Finding of relevance for this study
Depression prevalence							
Cobb, *et al*. (2014)	Not reported	*N* = 298	Boston, St. Louis, New York City, Iowa City and Chicago, USA	Cohort study	Semistructured interview	LIFE Psychiatric Status Rating scales ⩾ 3	Significant differences observed in *post hoc* test defining winter from December through April (*p* = 0.011). Relapse and onset more likely in winter months.
de Graaf *et al*. (2005)	February 1996–January 1997	*N* = 7076	Netherlands	Repeated cross-sectional measurements	CIDI (Composite international diagnostic interview)	DSM-III-R criteria for mood disorders.	No difference
Doganer *et al*. (2015)	Not reported	*N* = 2873	Rochester, Minnesota, USA	Cohort study	PHQ-9 (Patient Health Questionnaire – 9)	Remission of depression, definition not presented.	A higher proportion of the participants (26.9%) were first diagnosed in spring than during the winter (21.5%).
Huibers *et al*. (2010)	December 2005–December 2006	*N* = 14 478	Netherlands	Cohort study	DID (Diagnostic Interview for Depression)	DSM-IV criterion for MDD and DID ⩾ 2	Higher prevalence of MDD in summer compared to spring (*p* < 0.01) and autumn compared to spring (*p* < 0.01). Highest prevalence of reduced mood was found in autumn (*p* < 0.01).
Kristjánsdóttir *et al*. (2013)	August 2005–July 2006	*N* = 1250	Uppsala, Sweden	Repeated cross-sectional measurements	SF-36 (Short Form – 36) and MADRS-S (Montgomery Aasberg Depression Rating Scale)	SF-36: MH ⩽ 48 og VT ⩽ 40 MADRS-S ⩾ 11 and MADRS-S ⩾20	A higher proportion scored over cut-off on MADRS-S in January (46%) *v*. June (24%). Proportion scoring moderate depressive episode was 13–18% in January *v*. 5–6% in July (*p* < 0.05). On VT subscale, a higher proportion (*p* < 0.001) of participants scored over cut-off in November–January (from 43 to 53%) compared to July to August (16 and 19%). MH subscale higher proportion in November (40%) and December (38%) compared to July (17%) and August (14%) (*p* < 0.001). Higher proportion with depression in May (36%) compared to July and August (*p* < 0.0001).
Michalak *et al*. (2004)	November 1996–December 2007	*N*(UK) = 1299 *N*(Finland) = 1352 *N*(Norway) = 2711 *N* (Spain) = 1246	Great Britain, Norway, Spain and Finland	Repeated cross-sectional measurements	BDI (Beck Depression Interview)	BDI ⩾ 13	No difference
Murase *et al*. (1995)	Not reported	*N* = 161	Stockholm, Sweden	Repeated measurements and cohort study	BDI (Beck Depression Interview)	BDI ⩾ 10	Higher prevalence in winter compared to summer (*p* < 0.05).
Patten *et al*. (2017)	1996–2013	*N* = 516 911	Canada	Repeated cross-sectional measurements	CIDI-SFMD (Composite International Diagnostic Interview – Short Form Major Depression)	CIDI-SFMD ⩾ 5	Highest prevalence in winter and lowest in summer (*p* < 0.001). No difference in latitude.
Stordal *et al*. (2008)	August 1995–June 1997	*N* = 60 995	Nord-Trøndelag, Norway	Repeated cross-sectional measurements	HADS (Hospital Anxiety and Depression Scale)	HADS-D ⩾ 8	Overall trend (*p* < 0.001), with lowest prevalence in May and highest in January.
Traffanstedt *et al*. (2016)	2006	*N* = 34 294	USA	Repeated cross-sectional measurements	PHQ-8 (Patient Health Questionnaire – 8)	PHQ-8 Days ⩾ 55	No difference
Depression symptoms							
Albin (1982)	Not reported	*N* = 160	Boston, USA	Repeated measurement design	CES-D (Center for Epidemiologic Studies – Depression)	CES-D scores	No difference.
de Craen *et al*. (2005)	1997–1999	*N* = 500	Leiden, Netherlands	Repeated measurement design	GDS-15 (Geriatric Depression Scale)	GDS-15-scores	No difference.
Harris and Dawson-Hughes (1993)	1989	*N* = 250	Boston, USA	Repeated measurement design	POMS (Profile of Mood States)	POMS-scores	Those measured in August and September had lower levels of tension anxiety (*p* = 0.039), *Depression-Dejection* (*p* = 0.032), *Anger-Hostility* (*p* < 0.001), and *Confusion-Bewilderment* (*p* = 0.0043) compared to participants measured in October or November.
Kerr *et al*. (2013)	1:1984–2006 2: 1989–2008	N1 = 206 N2: 559	1: USA, Oregon 2: USA, Iowa	Repeated measurement design	1: CES-D (Center for Epidemiologic Studies – Depression) 2: SCL-90-D (Symptom Checklist – 90)	1: CES-D 22 for adolescents, 16 for adults 2: SCL-90-D above 90^th^ percentile	Two longitudinal cohorts followed up from 10–19 times with *n* = 8316 person observations. Modest probability of clinically relevant symptoms in early winter.
Magnusson *et al*. (2000)	January, April, July, October 1989	*N* = 2262	Iceland	Repeated cross-sectional survey	HADS (Hospital Anxiety and Depression Scale)	Mean scores on continuous scale	No difference in mean scores between the measurement time points
O'Hare *et al*. (2016)	2009–2011	*N* = 8027	Ireland	Cross-sectional survey (part of a prospective cohort of age 50+)	CES-D (Center for Epidemiologic Studies – Depression)	CES-D score	Significantly higher CES-D score (6.56 (6.09, 7.04)) in winter compared to spring (5.81(5.40, 6.22)) and autumn (5.82(5.36, 6.26)). However, not summer (6.00(5.48, 6.52))
Park *et al*. (2007)	Not reported	*N*(Rochester) = 24 *N*(San Diego) = 30	Rochester, Minnesota and San Diego, California, USA	Repeated measurement design	CES-D (Center for Epidemiologic Studies – Depression) SIGH-SAD	CES-D-scores and SIGH-SAD-scores	Higher CES-D-and SIGH-SAD scores in winter compared to summer in Rochester sample (*p* < 0.038 and *p* < 0.009). No difference in San Diego sample.
Schlager *et al*. (1993)	October 1987 –August 1988	*N* = 1870 (1556 male, 314 female)	Pennsylvania, USA	Cross-sectional survey	HSCL (Hopkins Symptom Checklist)	1:Expanded mood scale (mean difference) 2:HSCL score 1 s.d. above annual mean	Higher symptom levels in autumn and winter in women (EMS: *F* = 2.83, *p* < 0.05, HSCL depression: *r* = −0.1, *p* < 0.01), but not men.
Winthorst *et al*. (2011)	January 2004–February 2007	N1 = 5549 N2 = 1090	Amsterdam and Groningen, Netherlands	Repeated measurement design	1:K-10 (Kessler-10) 2:IDS (Inventory of Depressive Symptomatology) and BAI (Beck Anxiety Inventory)	1:K-10 scores 2:IDS & BAI-scores	1:No difference 2:No difference
Papers on post-natal depression							
Ballard *et al*. (1993)	Not specified	*N* = 28	Coventry, England	Repeated cross-sectional study	PAS (Psychiatric Assessment Schedule)	PAS/RDC-criterion for post-natal depression	Higher prevalence in autumn (*n* = 12) compared to in spring (*n* = 6) (*p* < 0.001).
Henriksson *et al*. (2017)	2010–2015	*N* = 4129	Uppsala, Sweden	Nested case-control study, participants in a population-based cohort (BASIC) who gave birth at a single hospital site 2010–2015.	EPND (Edinburgh postnatal depression scale)	EPND score >12	No seasonal pattern was observed comparing Oct0ber-December births with April-June; increased winter symptoms in one of four years only.
Jewell *et al*. (2010)	2004–2006	*N* = 67 079	16 of the 37 US states participating in PRAMS.	Population-based dataset exploring attitudes and experiences before, during and after birth in 37 US states.	PHQ-2 (Patient Health Questionnaire, modified, included in Pregnancy Risk Assessment Monitoring System (PRAMS))	PHQ-2 score ⩾ 5 for depression and ⩾ 3 for mild/subthreshold depression.	No relationship between mild or moderate post-partum depression and either season of birth or daylight length at time of birth.
Sit *et al*. (2011)	2006–2010	*N* = 9339	Allegheny County, Pennsylvania, USA	Repeated cross-sectional study	EPDS (Edinburgh postnatal depression scale)	EPDS/EPDS ⩾ 10	Prevalence lowest in June (96/827 = 11.6%) and July (94/751 = 2.5%), and highest in November (153/928 = 16.5%) and December (132/824 = 16.0%).
Sylvén *et al*. (2011)	May 2006–June 2007	*N* = 2318	Uppsala, Sweden	Cohort study	EPDS (Edinburgh postnatal depression scale)	EPDS/EPDS ⩾ 11.5	Higher rate of post-natal depression among women who gave birth in fourth quartal, 6 weeks (OR = 2.02, 1.32–3.10) and 6 months (OR = 1.82, 1.15–2.88) after giving birth.
Weobong *et al*. (2015)	March 2008–July 2009	*N* = 13 360	Brong Ahafo, Ghana	Cohort study	PHQ-9 (Patient Health Questionnaire – 9)	PHQ-9/PHQ-9 ⩾ 5	Mothers who gave birth during drought season had higher risk of depression compared to those who gave birth during rain-season (*p* = 0.006).
Yang *et al*. (2011)	2005	*N* = 2107	Taiwan	Registry	National health research database, Taiwan.	ICD-9-CM criterion for post-natal depression.	Highest prevalence of post-natal depression among those who gave birth during winter (23.93%), lowest during summer (16.82%) (*p* < 0.0001).
Antidepressant medication							
Balestrieriet *et al*. (1991)	1983–1988	Not reported	Verona, Italy	Registry	Prescription database	DDD	Highest proportion of antidepressants prescribed in spring with highest rates in May.
Gardarsdottir *et al*. (2010)	2002–2007	*N* = 16 289	Netherlands	Registry	Prescription database	N patients with incident prescription per season.	Higher rate of incident prescription in winter compared to summer (*p* < 0.01).
Skegg *et al*. (1986)	June 1974–February 1976	*N* = 2077	Oxford and Worcestershire, England	Registry	Prescription database	Antidepressant prescription	For males, a higher rate of prescriptions in June and December. No difference for women.
Admissions and care							
Anastasi, *et al*. (2014)	July 2011–June 2012	*N* = 675	Perugia, Italia	Registry	Clinical interview	ICD-10 depression	Highest prevalence in February and august (0.89%). Lowest in October (0.15%), November (0.15%) and December (0.15%).
Belleville *et al*. (2013)	March 2005–April 2008	*N* = 771	Lévis, Canada, and Montreal, Canada	Repeated cross-sectional	ADIS (Anxiety Disorders Interview Schedule)	DSM-IV criteria for mood disorders.	No differences
Cerbus and Dallara (1975)	1971–1972	*N* = 115	Cincinnati, USA	Registry	Hospital admission registry	Depression.	No difference
Christensen *et al*. (1983)	1979–1981	*N* = 3517	Anchorage, Alaska	Registry	Data from emergency phone registry	Calls categorised as for depression	No difference.
Dominiak *et al*. (2015)	2002–2010	*N* = 681 recurrent depression; *N* = 909 single depressive episode; *N* = 131 bipolar depression	Warsaw, Poland	Registry	Psychiatric hospital admissions	Clinical diagnosis on discharge	ARIMA analysis of time series by diagnosis and gender was significant for seasonality by monthly time points but in no clear pattern.
Eastwood and Stiasny (1978)	1969–1974	Unknown	Ontario, Canada	Registry	Data from health registry	ICDA-8-criteria classified as endogenous and neurotic depression	Higher prevalence of endogenous depression in spring compared to winter (*p* < 0.001). Higher prevalence of neurotic depression in autumn compared to summer (*p* < 0.001).
Harris (1984)	1980	*N* = 3191	London, UK	Registry	Clinical interview	ICD-9-criteria for depression	Higher number of consultancies for depression per day in May and June, and November, December and January.
Holloway and Evans (2014)	2007, 2009 and 2011	Not presented	London, England	Registry	Data from referrals for psychiatric care for elderly.	Mentioning of depression, low mood, suicide, bipolar in referrals	No difference.
Posternak and Zimmerman (2002)	1995–2001	*N* = 15 000	Rhode Island, USA	Registry	Data from referrals to psychiatric care	Depression rates	No difference in onset of major depression or depressive symptoms in spring or winter.
Rollnik *et al*. (2000)	July 1991–June 1993	*N* = 3963	San Diego, USA	Registry	Clinical interview	DSM-III-R criterion for affective disorders	Highest prevalence of affective disorders in spring (27.8%) and lowest in autumn (22.7%) (*χ*^2^ = 20.98, df = 3, *p* < 0.0001).
Sato *et al*. (2006)	1995–2000	*N*(total) = 958 *N*(bipolar) = 95 *N*(unipolar depression) = 863 *N*(unipolar depression with DMX) = 77 *N*(unipolar depression without DMX) = 786	Munich, Germany	Registry	Interview with patients and next of kin	ICD-10 criterion for MDE	No difference in entire sample. No difference for unipolar depression, but indications of seasonality for unipolar depression without DMX (K-S = 1.98, *p* < 0.01) with highest prevalence in spring and lowest in autumn. For unipolar depression with DMX, prevalence was highest in autumn (K-S = 2.54, *p* < 0.01).
Szabo and Blanche (1995)	1989	*N* = 139	Johannesburg, South-Africa	Registry	Diagnoses based on journal data.	DSM III-R criteria for mood disorders.	Admissions for mood disorders more prevalent in winter (*n* = 48) and spring (*n* = 43), and lowest in autumn (*n* = 15) (*χ*^2^ = 18.32, df = 3, *p* < 0.01)

**Table 2.** Study quality assessment through an adapted version of the Newcastle-Ottawa Scale (NOS)

Group	Author	Selection	Comparability	Outcome	NOS-score
Postpartum depression	Ballard *et al*. (1993)	*	*	*	***
	Henriksson *et al*. (2017)	**	*	**	*****
	Jewell *et al*. (2010)	**	**	**	******
	Sit *et al*. (2011)	**	**	*	*****
	Sylvén *et al*. (2011)	**	**	**	******
	Weobong *et al*. (2015)	**	**	**	******
	Yang *et al*. (2011)	**	*	***	******
Admissions and care	Anastasi *et al*. (2014)	*		**	***
	Belleville *et al*. (2013)	*		**	***
	Cerbus and Dallara (1975)	**		**	****
	Christensen and Dowrick (1983)	**		**	****
	Dominiak *et al*. (2015)	**	**	**	******
	Eastwood and Stiasny (1978)	**	**	***	*******
	Harris (1984)	*	**	**	******
	Holloway and Evans (2014)	**	*	***	******
	Posternak and Zimmerman (2002)	*	**	***	******
	Rollnik *et al*. (2000)	**	*	***	******
	Sato *et al*. (2006)	*	**	***	******
	Szabo and Blanche (1995)	**		**	****
Antidepressant medication	Balestrieri *et al*. (1991)	**		***	*****
	Gardarsdottir *et al*. (2010)	**	**	***	*******
	Skegg *et al*. (1986)	**	**	***	*******
Depression symptoms	Albin (1982)	*	**	**	*****
	de Craen *et al*. (2005)	**	**	***	*******
	Harris and Dawson-Hughes (1993)	*	**	**	*****
	Kerr *et al*. (2013)	*	**	***	******
	Magnusson *et al*. (2000)	**	**	**	******
	O'Hare *et al*. (2016)	**	**	*	*****
	Park *et al*. (2007)		**	**	****
	Schlager *et al*. (1993)	**	*	**	*****
	Winthorst *et al*. (2011)	**	**	**	******
Depression prevalence	Cobb, *et al*. (2014)	**	**	***	*******
	de Graaf, *et al*. (2005)	**	**	***	*******
	Doganer *et al*. (2015)	**	**	*	*****
	Huibers, *et al*. (2010)	**	**	*	*****
	Kristjánsdóttir *et al*. (2013)	**	**	**	******
	Michalak *et al*. (2004)	**	**	**	******
	Murase *et al*. (1995)	*	**	**	*****
	Patten *et al.* (2017)	*	**	***	******
	Stordal *et al*. (2008)	**	**	**	******
	Traffanstedt *et al*. (2016)	**	**	**	******

The studies were sorted in five categories defined by study content (Table 2): The first comprised ten studies on *prevalence of depression*. Six of these were cross-sectional studies with data collections that spanned across seasons, four were cohort studies of which one used a repeated measurement design. Five of the studies (Murase *et al*., 1995; Stordal *et al*., 2008; Kristjansdottir *et al*., 2013; Cobb *et al*., 2014; Patten *et al*., 2017) observed indications of seasonality with higher prevalence in winter compared to summer. Notably, Patten *et al*. (2017) pooled data from ten surveys in Canada where depression was measured through standardised clinical interviews and found higher prevalence rates in the winter months. In Cobb *et al*. (2014), indications of seasonality was found in a *post hoc* test where winter was defined as lasting from December through April. Huibers *et al*. (2010) found indications of seasonality in depression, but with the highest prevalence in summer and autumn compared to spring. The study by Doganer *et al*. (2015) primarily focused on 6-month remission rates, but in their clinical sample, a higher rate were diagnosed in spring (26.9%) *v*. winter (21.5%). Three of the studies (Michalak *et al*., 2004; De Graaf *et al*., 2005; Traffanstedt *et al*., 2016) found no indications of seasonality.

Nine studies were sorted under *depressive symptoms*, all based on self-reported symptom levels through the use of questionnaires. Six of the studies used repeated measurement designs while three studies were single cross-sectional surveys spanning a year. In four of them, no indications of seasonality were found (Albin, 1982; Magnusson *et al*., 2000; De Craen *et al*., 2005; Winthorst *et al*., 2011). Park *et al*. (2007) found higher mean scores on CES-D during winter in a subsample, while Harris and Dawson-Hughes (1993) found higher levels of depressive symptoms in October and November compared to August and September. Schlager *et al*. (1993) found seasonal variation among women with a variety of symptoms elevated in winter, but no similar variation in men. O'hare *et al*. (2016) reported a cohort study in Ireland in which on a single cross-sectional measure, depression scores in autumn and spring only were lower than winter (summer scores were not significantly different). Kerr *et al*. (2013) followed two independent cohorts from school age into adulthood with 10–19 measurements (8316 person observations). In both samples, they observed a modest increase in depressive symptoms in winter, but no effect on caseness.

Seven studies covered *postpartum depression*, thus consisting of populations that recently have given birth. The most common design in this group were studies with repeated cross-sectional measurements, and most common symptoms were assessed with the Edinburgh Postnatal Depression Scale (Cox *et al*., 1987). In four of these studies, the prevalence of depressive symptoms was higher among mothers who gave birth in winter/autumn (Ballard *et al*., 1993; Sit *et al*., 2011; Sylven *et al*., 2011; Yang *et al*., 2011). Henriksson *et al*. (2017) reported no overall association in Swedish mothers at one hospital. Jewell *et al*. (2010) used a large sample from the US PRAMS dataset and found no indications of seasonal variation in postpartum depression. In the final study in this group, Weobong *et al*. (2015) found a higher prevalence of depressive symptoms in the drought season compared to the rainy season of Ghana (near the equator).

Two sets of studies focussed on health care use. Three studies used registry data on *antidepressant prescriptions*. All these three observed seasonal patterns; Balestrieri *et al*. (1991) found more prescriptions in autumn and spring. Skegg *et al*. (1986) found a higher rate in December and June in men but not women, while the last study by Gardarsdottir *et al*. (2010) found more prescriptions in winter. Twelve studies addressed aspects of *admissions and care* based on individual contact with health services. With the exception of Belleville *et al*. (2013), all were registry studies. Six of them (Cerbus and Dallara, 1975; Christensen and Dowrick, 1983; Posternak and Zimmerman, 2002; Belleville *et al*., 2013; Holloway and Evans, 2014) found no indications of seasonality, including Sato *et al*. (2006) that found no overall association, but higher rates of prescriptions for major depressive episode in spring among individuals with unipolar depression without depressed mixed states, and in autumn for bipolar and unipolar individuals with depressed mixed states. Szabo and Blanche (1995) found more admissions for mood disorder in winter. The remaining five studies in this group found indications of seasonality, but not in winter (Eastwood and Stiasny, 1978; Harris, 1984; Rollnik *et al*., 2000; Anastasi *et al*., 2014; Dominiak *et al*., 2015).

## Discussion

### Main finding

The main purpose of this study was to review the question of seasonality of depression excluding studies with high risk of bias through subjective reporting. Of 41 studies, 13 had a main conclusion that suggested more depression in winter (Table 3). The remaining studies either suggested no seasonal pattern, indications of seasonality but outside winter, or ambiguous results in terms of seasonality. The total evidence across the studies was highly equivocal with great heterogeneity in both research questions addressed, study design, definition of seasons, data collection, and statistical analysis. The results were not uniform across the studies, and it is not clear which months are implicated and how to define the season with increased risk. Half of the included studies on depression prevalence found results in line with seasonality in clinical depression. Beyond a possible impact of seasonality on clinical depression, we did not find convincing evidence for seasonality effect in depressive symptoms at the population level.

**Table 3.** Crude classification of number of papers with main result suggesting no seasonality, winter seasonality, other seasonality or ambiguous results in each of the study categories

Study category	# of papers suggesting no seasonality	# of papers suggesting increased depression in winter	# of papers with seasonal effects outside winter or ambiguous results
Depression prevalence	3	5	2
Depression symptoms	4	2	3
Post-natal depression	2	4	1
Antidepressive medication	0	1	2
Admissions and care	6	1	5
Sum	15	13	13

### Strengths and limitations

The main strength of this study was the systematic approach to search and appraise the literature with design constraints to minimise risk of bias. The broad search strategy could be both a strength and a limitation, but the opportunity to review adjacent aspects of depression together could be of value given the scattered literature on this topic.

We did not register a protocol for this review in advance, which is a limitation. The large heterogeneity of studies, data, and designs restricted us from conducting meta-analyses. It also precluded any approximation of the impact of publication bias, which typically results in non-conservative results (i.e. studies that support the associations of interest are more likely to appear in the published literature) (Dwan *et al*., 2013). Any bias that increases the likelihood of studies with no difference across seasons to remain unpublished would weaken the empirical support for seasonality of depression. Due to heterogeneity between study designs and reporting it was a challenge to find a standard tool to assess study quality. We ended up with adapting an existing framework (NOS), but assessment and analysis of study quality remained difficult due to the range of approaches used in this literature. Finally, study search and selection was challenging due to study heterogeneity and the broad scope we set up for this search. Some of the included studies were found through in additional searches and reference lists and additional relevant data and studies not identified by us may exist. Our scope for this review did not include careful differentiation between depression subtypes such as unipolar or bipolar depression.

### Interpretation

There was a notable lack of consistency of effect in several studies observing seasonal effects. Skegg *et al*. (1986) found a difference for males only and only after adjusting for a declining time-trend in antidepressant use. Schlager *et al*. (1993) found differences for women but no difference for men. Cobb *et al*. (2014) found the difference in a *post hoc* test after the definition of winter was extended to include April, and Huibers *et al*. (2010) found increased rates in summer and autumn. Park *et al*. (2007) found a trend in only one of two samples. The large study from Patten *et al*. (2017) used a diagnostic interview to identify depression but still relied on subjective recall of onset, with some inherent risk of memory bias. Kerr *et al*. (2013) used within-subjects repeated measurements. Although they found indications of more depressive symptoms in winter, effect sizes were minute. Many of the studies reported prevalence rates by month, rather than incidence rates that arguably are better suited to inform causal hypotheses on season and illness onset.

Four of seven studies on post-natal depression presented seasonal differences with higher prevalence among mothers who gave birth in autumn/winter compared to spring and summer. Biological causal models, often based on daylight deprivation, are frequently proposed. Social factors might also be of relevance and can coincide and/or reinforce with biological factors. For example, lack of social support is an acknowledged risk factor for postpartum depression (Kim *et al*., 2014) and availability of social support could vary with seasons due to fewer outdoor activities or seasonal work patterns.

The studies on antidepressant prescriptions all observed seasonal variation, and two of them found the highest prescription rates in winter. These studies have high internal validity in that they present objective data with accurate dates, but they also reflect a response to illness rather than incidence of depression itself. Increased prescription rates can be a result of more severe episodes of clinical depression during the winter which increases both help-seeking and treatment response during those periods. It is also possible that some GPs more readily attribute symptom presentations to depression during certain seasons, which could also contribute to increased prescription.

The literature on seasonality of depressive illness have frequently cited access to daylight as a plausible mechanism, based on the phase shift hypothesis (Lewy *et al*., 1987) and the latitude hypothesis (Potkin *et al*., 1986). Melatonin levels correlate negatively with light stimulus and promotes drowsiness (Srinivasan *et al*., 2006). It is suggested that light deprivation brings on seasonal phase shifts in hormone levels, with Melatonin particularly implicated, which in turn may increase the risk of depression. Our results do not provide any clear support to this hypothesis as no clear population level trend was found and reiterates results of previous reviews of this question (Mersch *et al*., 1999). The latitude hypothesis and reduced daylight access have also given rise to light-therapy as intervention, but the evidence for its efficacy in preventing depression remains limited (Nussbaumer *et al*., 2015).

This systematic review did not point towards a clear and unified pattern on seasonal variation in depression and depressive symptoms. This does not exclude that seasonal variation influences individuals. Neither does it exclude that for some, such variation may shift individuals to clinically relevant states. It is possible that environmental seasonal change to some extent affects everyone, but that we cope and adapt in ways embedded in culture, behavioural patterns, technology, and societal structures. As exemplified by Kerr *et al*. (2013), other risk factors for depression seem more salient.

Our results are relevant for the longstanding discussion around seasonal affective disorder. Some of the studies included here did point to a change in the prevalence of depression with seasons. However, we do not see the results from the studies included in this review to be in support of any strong general and public health relevant effect of seasons on mood.

### Suggestions for future research in this field

The identified studies used highly heterogeneous study designs and the fragmented results suggest a potential for methodological improvements in this research. The many ways to measure and operationalise depression was also reflected here in terms of scales used, cut-offs and case definitions. Regarding measurement density, some studies had two measurements over the course of a year, while others had monthly registrations. There was also little consensus as to how seasons or winter was defined across studies. Some examined specific months while others used broader categories such as spring and autumn. For example, Cobb *et al*. (2014) included April in winter, while Michalak *et al*. (2004) defined April as part of spring. Yet others defined seasons in relation to winter and summer solstice and in many studies definitions of seasons remained unclear.

Many of the studies included in this review used cross-sectional data collections that ran over time and covered the seasons of interest, but that was set up for other purposes than to study seasonality. This design ensures that participants were indeed blind to the research hypothesis. A disadvantage is that design features, such as choice of measurement, timing and frequency seemed less than optimal for many of the papers. For many of the cross-sectional data collections, it was unknown when cases had their onset. As such, cases identified at a given time point may both reflect increased incidence at that time, but also reduced remission rates. This challenges interpretations.

Our results suggest there is a need for more high quality, unbiased studies on seasonal variation in depression. Nominal exposure categories such as ‘winter’ is a crude term to describe exposures, and future studies should accurately state the time-period definitions coupled with informative data on the assumed underlying mechanism. Where possible, analyses should include geographical data and other contexts that could relate to observations such as climate and weather. There may also be important confounders to consider, such as physical activity, sleep and food intake that could both be confounders but also potential mechanisms between season and mental health. Clinical registry data could provide an excellent data source by providing incidence rates per time. Repeated surveys with screening tools will most often reflect prevalence, which could both be a derivative of seasonal variation in remission rates as well as seasonal onset. Precision around these features of studies is important for interpretation and allow for meta-analysis in future reviews in this area.

## Conclusion

We conclude that there is some support for seasonal variation in clinical depression, but that this is not likely due to a broad and general mechanism where entire populations are shifted towards lower mood and more sub-threshold symptoms at regular intervals throughout the year. This could be an important nuance for the public, particularly those exposed to major shifts in daylight that frequently get information that suggest winter and less daylight will bring down your mood. Further development in this field will require higher study quality and more unbiased population-based studies on the potential relationship between seasonal changes and depression.

## Availability of data and materials

All data for this review are available in the included papers. Details on quality assessment of single studies is available upon request to the corresponding author.

## Appendix

### Search terms used for: seasonality and symptoms of depression: a systematic review of the literature

#### Approach

The following databases were accessed during the electronic component of the systematic review: Cochrane Database of Systematic Reviews, DARE (Database of Abstracts of Reviews of Effects), Cochrane Central Register of Controlled Trials (CENTRAL), Medline and Medline in Process (via OVID), Embase (via OVID), PsycINFO (via OVID) and the Cumulative Index to Nursing and Allied Health Literature (CINAHL). A search strategy was developed to identify keywords and Medical Subject Headings (MeSH) in Medline, which were then adapted for all other databases.

=2121

#### Medline in Process and OVID Medline

1. Seasons/
2. (season* adj3 varia*).tw.
3. seasonalit*.tw.
4. (season* adj2 pattern*).tw.
5. (periodic* adj3 varia*).tw.
6. (periodic* adj3 fluctuation*).tw.
7. (season* adj2 adjust*).tw.
8. (season* adj2 change*).tw.
9. (season* adj2 rhythm*).tw.
10. (season* adj2 inciden*).tw.
11. or/1-10
12. Depression/
13. exp Self-Injurious Behavior/
14. exp Anxiety/
15. exp Anxiety Disorders/
16. exp Mood Disorders/
17. exp Sleep Disorders/
18. depress*.tw.sea
19. parasuicide*.tw.
20. automutilation.tw.
21. (self adj1 harm*).tw.
22. (self adj1 destruct*).tw.
23. (self adj1 injur*).tw.
24. (self adj1 mutilat*).tw.
25. suicid*.tw.
26. anxiet*.tw.
27. nervousness.tw.
28. hypervigilance*.tw.
29. (anxi* adj3 (dis* or syndrome* or neuros#s)).tw.
30. (personalit* adj2 anankastic*).tw.
31. agoraphob*.tw.
32. (panic adj2 (disorder* or attack*)).tw.
33. hoard*.tw.
34. (phobia adj2 disorder*).tw.
35. (stress adj3 disorder*).tw.
36. (hyperkinetic adj2 heart adj2 syndrome*).tw.
37. (neuros#s adj2 cardiac*).tw.
38. (effort* adj2 syndrome*).tw.
39. (neurocirculator* adj2 asthenia*).tw.
40. (affective adj2 (disorder* or psychos#s)).tw.
41. (mood adj1 disorder*).tw.
42. (bipolar adj2 disorder*).tw.
43. manic-depressi*.tw.
44. melancholi*.tw.
45. (cyclothymic* adj2 disorder*).tw.
46. insomn*.tw.
47. (sleep adj2 (dis* or dysfunction* or syndrome* or deprivation or paroxysm* or myoclonu* or hyponea* or apnea*)).tw.
48. dyssomnia*.tw.
49. hypersomnia*.tw.
50. (excessiv* adj2 somnolence*).tw.
51. hypersomnolence*.tw.
52. narcoleps*.tw.
53. (gelineau adj1 syndrome*).tw.
54. catalep*.tw.
55. (kleine-levin adj1 syndrome*).tw.
56. (toneless* adj1 syndrome*).tw.
57. (nocturnal adj2 myoclon*).tw.
58. (henneberg adj1 syndrome*).tw.
59. (restless adj3 syndrome*).tw.
60. (periodic adj3 movement adj1 disorder*).tw.
61. (willis-ekbom adj1 (dis* or syndrome*)).tw.
62. (wittmaack-ekbom adj1 (dis* or syndrome*)).tw.
63. (apnea adj2 central*).tw.
64. (central adj3 hypoventilation*).tw.
65. (ondine adj2 syndrome*).tw.
66. (pickwickian adj2 syndrome*).tw.
67. or/12-66
68. exp Adult/
69. Adolescent/
70. adult*.tw.
71. adolescent*.tw.
72. teen*.tw.
73. or/68-72
74. 11 and 67 and 73

#### Embase

1. exp season/
2. (season* adj3 varia*).tw.
3. (season* adj2 pattern*).tw.
4. (periodic* adj3 varia*).tw.
5. (periodic* adj3 fluctuation*).tw.
6. (season* adj2 adjust*).tw.
7. (season* adj2 change*).tw.
8. (season* adj2 rhythm*).tw.
9. (season* adj2 inciden*).tw.
10. seasonalit*.tw.
11. or/1-10
12. exp depression/
13. automutilation/
14. anxiety/
15. exp anxiety disorder/
16. exp mood disorder/
17. sleep disorder/
18. depression.tw.
19. parasuicide*.tw.
20. (self adj1 harm*).tw.
21. (self adj1 destruct*).tw.
22. (self adj1 injur*).tw.
23. (self adj1 mutilat*).tw.
24. suicid*.tw.
25. anxiet*.tw.
26. nervousness.tw.
27. hypervigilance*.tw.
28. (anxi* adj3 (dis* or syndrome* or neuros#s)).tw.
29. (personalit* adj2 anankastic*).tw.
30. (panic adj2 (disorder* or attack*)).tw.
31. agoraphob*.tw.
32. hoarder*.tw.
33. (phobia adj2 disorder*).tw.
34. (stress adj3 disorder*).tw.
35. (hyperkinetic adj2 heart adj2 syndrome*).tw.
36. (neuros#s adj2 cardiac*).tw.
37. (effort* adj2 syndrome*).tw.
38. (neurocirculator* adj2 asthenia*).tw.
39. (affective adj2 (disorder* or psychos#s)).tw.
40. (mood adj1 disorder*).tw.
41. (bipolar adj2 disorder*).tw.
42. manic-depressi*.tw.
43. melancholi*.tw.
44. (cyclothymic* adj2 disorder*).tw.
45. insomn*.tw.
46. (sleep adj2 (dis* or dysfunction* or syndrome* or deprivation or paroxysm* or myoclonu* or hyponea* or apnea*)).tw.
47. dyssomnia*.tw.
48. hypersomnia*.tw.
49. (excessiv* adj2 somnolence*).tw.
50. hypersomnolence*.tw.
51. narcoleps*.tw.
52. (gelineau adj1 syndrome*).tw.
53. catalep*.tw.
54. (kleine-levin adj1 syndrome*).tw.
55. (toneless* adj1 syndrome*).tw.
56. (nocturnal adj2 myoclon*).tw.
57. (henneberg adj1 syndrome*).tw.
58. (restless adj3 syndrome*).tw.
59. (periodic adj3 movement adj1 disorder*).tw.
60. (willis-ekbom adj1 (dis* or syndrome*)).tw.
61. (wittmaack-ekbom adj1 (dis* or syndrome*)).tw.
62. (apnea adj2 central*).tw.
63. (central adj3 hypoventilation*).tw.
64. (ondine adj2 syndrome*).tw.
65. (pickwickian adj2 syndrome*).tw.
66. or/12-65
67. adult/
68. adolescent/
69. adult*.tw.
70. adolescent*.tw.
71. teen*.tw.
72. or/67-71
73. 11 and 66 and 72

#### PsycINFO

1. seasonal variations/
2. seasonalit*.tw.
3. (season* adj2 adjust*).tw.
4. (season* adj2 change*).tw.
5. (season* adj2 inciden*).tw.
6. (season* adj2 pattern*).tw.
7. (season* adj2 rhythm*).tw.
8. (season* adj3 varia*).tw.
9. (periodic* adj3 fluctuation*).tw.
10. (periodic* adj3 varia*).tw.
11. or/1-10
12. exp affective disorders/
13. exp self destructive behavior/
14. exp anxiety/
15. exp anxiety disorders/
16. exp sleep disorders/
17. depress*.tw.
18. parasuicide*.tw.
19. automutilation.tw.
20. (self adj1 harm*).tw.
21. (self adj1 destruct*).tw.
22. (self adj1 injur*).tw.
23. (self adj1 mutilat*).tw.
24. suicid*.tw.
25. anxiet*.tw.
26. nervousness.tw.
27. hypervigilance*.tw.
28. (anxi* adj3 (dis* or syndrome* or neuros#s)).tw.
29. (personalit* adj2 anankastic*).tw.
30. agoraphob*.tw.
31. (panic adj2 (disorder* or attack*)).tw.
32. hoard*.tw.
33. (phobia adj2 disorder*).tw.
34. (stress adj3 disorder*).tw.
35. (hyperkinetic adj2 heart adj2 syndrome*).tw.
36. (neuros#s adj2 cardiac*).tw.
37. (effort* adj2 syndrome*).tw.
38. (neurocirculator* adj2 asthenia*).tw.
39. (affective adj2 (disorder* or psychos#s)).tw.
40. (mood adj1 disorder*).tw.
41. (bipolar adj2 disorder*).tw.
42. manic-depressi*.tw.
43. melancholi*.tw.
44. (cyclothymic* adj2 disorder*).tw.
45. insomn*.tw.
46. (sleep adj2 (dis* or dysfunction* or syndrome* or deprivation or paroxysm* or myoclonu* or hyponea* or apnea*)).tw.
47. dyssomnia*.tw.
48. hypersomnia*.tw.
49. (excessiv* adj2 somnolence*).tw.
50. hypersomnolence*.tw.
51. narcoleps*.tw.
52. (gelineau adj1 syndrome*).tw.
53. catalep*.tw.
54. (kleine-levin adj1 syndrome*).tw.
55. (toneless* adj1 syndrome*).tw.
56. (nocturnal adj2 myoclon*).tw.
57. (henneberg adj1 syndrome*).tw.
58. (restless adj3 syndrome*).tw.
59. (periodic adj3 movement adj1 disorder*).tw.
60. (willis-ekbom adj1 (dis* or syndrome*)).tw.
61. (wittmaack-ekbom adj1 (dis* or syndrome*)).tw.
62. (apnea adj2 central*).tw.
63. (central adj3 hypoventilation*).tw.
64. (ondine adj2 syndrome*).tw.
65. (pickwickian adj2 syndrome*).tw.
66. or/12-65
67. 11 and 66
68. limit 67 to (200 adolescence or ‘300 adulthood’)
